# Comparison of ATP binding sites using structure-based similarity methods and molecular interaction fields

**DOI:** 10.1186/1758-2946-3-S1-P34

**Published:** 2011-04-19

**Authors:** J Dreher, K Baumann

**Affiliations:** 1 Pharmaceutical Chemistry, University of Technology, Beethovenstr. 55, 38106 Braunschweig, Germany

## 

Protein kinases represent one of the largest group of drug targets in humans. All kinase enzymes share a catalytic domain that binds ATP. In recent years, many small molecule ATP-competitive kinase inhibitors have been developed. However, the evolutionary relatedness and structural conservation of these targets often lead to unforeseen cross reactivity.

The goal of this work was to develop structure based ATP binding site descriptors that reflect the pharmacological profile of a predefined set of inhibitor molecules. For this purpose, a 3-step procedure was applied. First, a multiple sequence alignment generated by Buzko and Shokat [1] was adopted and binding site residues were identified. Second, kinase crystal structures were collected from SwissProt searches with EC numbers and superimposed based on the aligned binding site C-alpha atoms. Finally, molecular interaction field (MIF) descriptors were calculated and compared using a fuzzy Dice coefficient modified for 3D Euclidean space. For reasons of comparability with MIF descriptors, sequence descriptors based on the alignment were calculated. Moreover, kinase-ligand interaction fingerprints based on the superimposition of co-crystallized Xâ€‘ray structures were computed and mapped on the alignment.

A dataset of 38 kinase inhibitors with experimental data against 90 human kinases was used to assess the performance of the developed descriptors [2]. Performance was measured by ROC enrichments for sampled kinase structures and Neighborhood Behavior criteria [3] for the complete kinase panel. Here, the performance is based on a comparison of the pairwise distances of the binding site descriptors to the pairwise distances of the inhibitor’s pharmacological profiles.

Overall, classification based on the sequence of the binding pocket performed best. However, sequence-based methods cannot detect unrelated kinases with similar pharmacological profile. This in turn is the strength of the MIF-based descriptors since these descriptors characterize the geometry of hot spots on the protein and are independent of sequence information. Visualization of binding site hot spots makes this method suitable to rationally optimize the selectivity profile of a compound.
